# The Digital Way to Intercept Psoriatic Arthritis

**DOI:** 10.3389/fmed.2021.792972

**Published:** 2021-11-23

**Authors:** Ivan Giovannini, Philipp Bosch, Christian Dejaco, Gabriele De Marco, Dennis McGonagle, Luca Quartuccio, Salvatore De Vita, Enzo Errichetti, Alen Zabotti

**Affiliations:** ^1^Rheumatology Clinic, Department of Medicine, University of Udine, c/o Azienda Sanitaria Universitaria Friuli Centrale, Udine, Italy; ^2^Department of Rheumatology and Immunology, Medical University of Graz, Graz, Austria; ^3^Department of Rheumatology, Hospital of Bruneck, Bruneck, Italy; ^4^Leeds Institute of Rheumatic and Musculoskeletal Medicine (LIRMM), University of Leeds, Leeds, United Kingdom; ^5^Department of Medical and Biological Sciences, Institute of Dermatology, University of Udine, Udine, Italy

**Keywords:** Psoriatic Arthritis (PsA), psoriasis, technology, prevention, interception, early diagnosis, telerheumatology

## Abstract

Psoriasis (PsO) and Psoriatic Arthritis (PsA) are chronic, immune-mediated diseases that share common etiopathogenetic pathways. Up to 30% of PsO patient may later develop PsA. In nearly 75% of cases, skin psoriatic lesions precede arthritic symptoms, typically 10 years prior to the onset of joint symptoms, while PsO diagnosis occurring after the onset of arthritis is described only in 15% of cases. Therefore, skin involvement offers to the rheumatologist a unique opportunity to study PsA in a very early phase, having a cohort of psoriatic “risk patients” that may develop the disease and may benefit from preventive treatment. Progression from PsO to PsA is often characterized by non-specific musculoskeletal symptoms, subclinical synovio-entheseal inflammation, and occasionally asymptomatic digital swelling such as painless toe dactylitis, that frequently go unnoticed, leading to diagnostic delay. The early diagnosis of PsA is crucial for initiating a treatment prior the development of significant and permanent joint damage. With the ongoing development of pharmacological treatments, early interception of PsA has become a priority, but many obstacles have been reported in daily routine. The introduction of digital technology in rheumatology may fill the gap in the physician-patient relationship, allowing more targeted monitoring of PsO patients. Digital technology includes telemedicine, virtual visits, electronic health record, wearable technology, mobile health, artificial intelligence, and machine learning. Overall, this digital revolution could lead to earlier PsA diagnosis, improved follow-up and disease control as well as maximizing the referral capacity of rheumatic centers.

## Introduction

Psoriasis (PsO) is a chronic, immune-mediated disease affecting 2–3% of the Caucasian population (1). Up to one third of PsO patients will eventually progress to Psoriatic Arthritis (PsA) [from 6 to 42% (2)], a chronic, inflammatory and potentially debilitating arthropathy (3). In nearly 75% of cases, psoriatic skin lesions precede arthritic symptoms, with an onset typically 10 years prior to joint symptoms, while PsO diagnosis occurring after the onset of arthritis is described only in 15% of PsA cases (4).

Progression from PsO to PsA most commonly evolves across several clinically silent stages (5), as well as the prodromal phase characterized by a short period of arthralgia and fatigue despite not having synovitis or enthesitis on physical examination, as reported by Scher and Zabotti (5–7). This gives the rheumatologist the unique window of opportunity to study PsA in a very early phase, having a cohort of psoriatic “risk patients” that may develop the disease and may benefit from preventive treatment. Severe PsO, nail involvement, familiarity for PsA, arthralgia and subclinical inflammation detected by imaging are considered predictors of PsA development in PsO patients (4, 7–10).

Early diagnosis of PsA is crucial for initiating a treatment prior the development of significant and permanent joint damage (11). The advances in PsA treatment in the latest decades have demonstrated a positive effect on prognosis and disability particularly in the initial phase of the disease, whereas diagnostic delay is associated with poorer outcome (12, 13). It is therefore crucial to identify PsO subjects at higher risk of developing PsA, who may benefit from early diagnosis and treatment, while there is a growing interest for preventive treatment.

Unfortunately, in daily routine of various health care systems around the world, the possibilities for the creation of a predictive models of PsA development are limited, and many obstacles have been reported (14).

The transition from PsO to PsA may go unnoticed or be undervalued, leading to a diagnostic delay and a poor window of treatment opportunity (13, 15). Furthermore, the lack of an adequate number of specialists further worsens access to rheumatologic services (16, 17), not only for diagnostic purposes, but also for follow-up. The visit frequency is so crucial for the management of the rheumatic disease, that EULAR treatment recommendations delineate the timing of the scheduled visits (18). In the course of rheumatic diseases, an adequate amount of monitoring visits for the assessment of disease activity is still challenging, even if frequent patient monitoring is an integral part of the treat-to-target strategy. In fact, frequent rheumatological visits are associated with improved outcomes (19), but due to the relapsing-remitting nature of the disease, an immediate rheumatological assessment in case of an acute exacerbation would be desirable. In practice, however, this is rarely feasible, and patients may be seen by the rheumatologist only after the disappearance of symptoms.

Additionally, the COVID-19 pandemic has led to radical changes in the management of rheumatic patients. The tangible risk of infection leads to a prolongation of monitoring intervals. During the first wave of the pandemic, some visits were postponed or canceled, with repercussions on patients' health, while others were replaced by telemedicine, in the form of phone calls and teleconferences (20, 21). In this scenario, the implementation of digitalization in rheumatology may represent an opportunity both for clinicians and patients (both PsO and PsA patients), as the technology may fill the gap between the demand for frequent monitoring visit and the limited resources to guarantee them. More details in Table 1.

**Table 1:** Digitalization in rheumatology.

**Digitalization in rheumatology: tools**					
**Virtual visits**	**Mobile health app**	**Wearable technology**	**Electronic health records**	**Artificial intelligence and machine learning**	**Digital therapeutics**
Phone-based calls	Self-assessment	Lifestyle tracker	Decision support	Symptom's checker	Treatment adherence
Video-assisted calls	Patient Reported Outcomes	Step counter	Information sharing	Classification of medical images	Self-injection device (e-device)
	Teledermatology	Wearable sensor	Records keeping	Prediction of complications	
	Remote patient monitoring			Risk stratification	

*Application of digital health technology in the transition from PsO to PsA setting*.

The aim of our review is to present insights on the digital approach in rheumatology, focusing on early diagnosis and follow-up in PsA.

### The Digital Approach Applied to Psoriatic Disease

In recent years, an extensive innovation in digitalization in rheumatology has occurred. The World Health Organization (WHO) defined “e-Health”, a collective term defined as “use of information and communications technology in support of health and health-related fields“ (14). This digital revolution included electronic health records, telemedicine and virtual visits, wearable technology, mobile health, artificial intelligence (AI), and machine learning.

The challenges in the development of digital health technologies are considerable, but the increased availability of these technologies offers the opportunity for improving clinical practice. These digital approaches have already been tested in rheumatology and may have great potential in the management of the transition phase of PsO to PsA. According to the author's experience, PsO patient can be stratified according to the increasing risk of developing PsA in three phases:

(i) **PsO patients at lower risk of developing PsA:** patients not presenting any known risk factors for progression to PsA.

(ii) **PsO** patients **at higher risk of developing PsA in a medium/long-term**: patients presenting known risk factors or predictors for progression to PsA, such as severe skin disease, nail involvement, familiarity for PsA (4, 8–10).

(iii) **PsO patients at higher and imminent risk of developing PsA**: patients presenting musculoskeletal complains suspicious for prodromal PsA, such as arthralgia, fatigue or Achilles' tendon pain (5, 7, 22).

**Figure 1:**
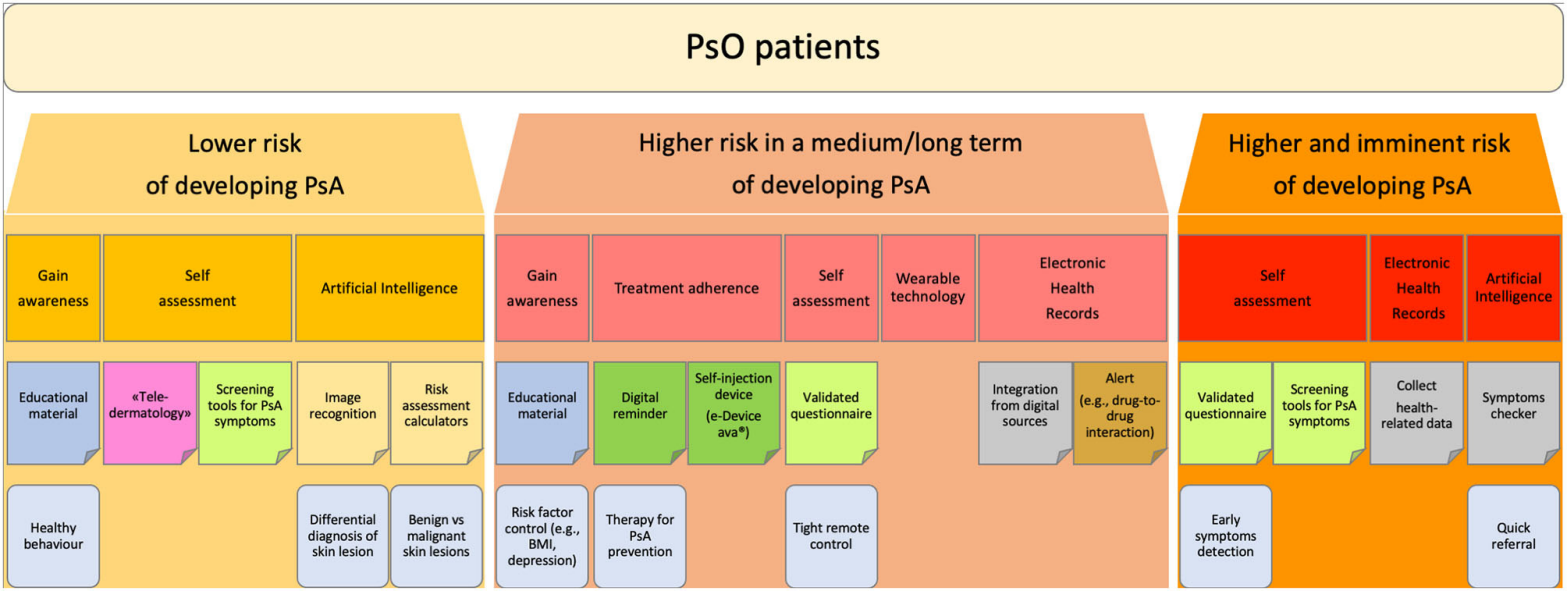
Flowchart: stratification of PsO patients. Proposed stratification of PsO patients according to the increasing risk of developing PsA.

A more comprehensive explanation of these group is addressed in the paragraph below (Figure 1).

The digitalization technology may offer a diversified set of digital tools which could offer new opportunities in the various PsO-to-PsA journey.

### PsO Patients at Lower Risk of Developing PsA

PsO patients with a lower risk of PsA represent the majority of PsO cases. They are usually not followed by dermatologists since they often present only with mild PsO treated with topic agents prescribed by the general practitioner (23–25). These patients nevertheless have a higher risk to develop PsA as compared to the general population (1).

PsO patients have to gain awareness of their own inflammatory disease and the comorbidities related to PsO. In fact, patients affected by PsO, even in the case of minimal skin lesions, exhibit not only an increased risk of developing PsA, but also display an higher risk of cardiometabolic disease, and endocrinological disease, such as diabetes mellitus (26–28). Therefore, the creation and distribution of educational material as well as awareness-raising campaigns, aiming to inform the patient about their own disease, spreading knowledge about the risks of developing PsA, promoting healthy behavior, and encouraging them to take health-related actions are highly beneficial (29). Informative campaign has already demonstrated efficacy in the management of rheumatological disease like gout (30–32), a known independent risk factor for PsA (33).

For these cases, digitalization may provide solutions. Digital “teledermatology” tools have been tested in dermatology for the self-assessment of psoriatic lesions. As proposed by Schreier et al., a smartphone app can be used by PsO patients with the aim of reporting active psoriatic lesions through pictures, leading to similar results as a face-to-face visits in the Psoriasis Area and Severity Index (PASI) and body surface area (BSA) assessment (34, 35). The self-assessment and monitoring of psoriatic lesions done by pilot teledermatology studies (36) may open the path to a remote monitoring of PsO patients who are not frequently evaluated by dermatologists, such as patients affected by minimal psoriasis, or mild psoriatic onychopathy.

The right moment when a rheumatologist should first see a PsO patient is still unclear. For PsO patients with a lower risk of PsA development, screening tools for PsA symptoms would be helpful for dermatologists and other physicians to better define the time point for a rheumatology visit. In axial spondyloarthritis (axSpA) several screening tools have already been proposed, such as the Berlin referral tool (37), which has shown to increase the probability of axSpA from 5% in patients with unselected chronic low back to 30–45% in case the tool yielded a positive result. Furthermore, screening for axSpA can also be performed online by patients with lower back pain increasing the proportion of positive axSpA from 5% (prevalence of the general population) to 19.4% (after triage with the online tool) (38). The recognition of patient with high probability of axial disease is of particular interest, because it may reduce the diagnostic delay in the diagnosis of axial involvement in PsA (39). Similar online screening tools could also be developed for PsO patients.

In the lower-risk subgroup of PsO patients, AI may also be helpful. The general aim of these technologies in health care is to assist clinical decision-making using computerized algorithms to uncover relevant information from big data (40), but also by learning from many sources (rather than being programmed with rules) including clinical, biological, and radiological data (41). At present, some pilot studies are testing machine learning in the dermatologic field, in particular in the analysis and classification of medical images, and prediction of complications (42). Other AI-based tools such as risk assessment calculators are becoming increasingly available, mostly focusing on differentiating benign and malignant skin lesions (43). Furthermore, Aggarwal (44) applied AI-machine learning for image recognition of various dermatological diseases, including PsO, reporting promising first results.

AI and machine learning have been of great interest not only in dermatology, but also in rheumatology. Guidelines for AI studies in rheumatology have been proposed by EULAR (45), and AI is currently being researched concerning its value for diagnosis, disease prediction, risk stratification, and monitoring of rheumatic diseases (46).

### PsO Patients at Higher Medium/Long-Term Risk of Developing PsA

PsO patients at higher risk of developing PsA in a medium/long-term are patients presenting with known risk factors or predictors for development to PsA. The identification of risk factors for PsA development in PsO patients is considered an unmet need in the EULAR recommendations (47).

In the years, the early diagnosis of PsA in a dermatological setting raised a lot of interest. Therefore, various screening tool for early identification of patients with PsA have been tested and validated, such as the Psoriasis Epidemiology Screening Tool (PEST) (48) and the Early ARthritis for Psoriatic patients (EARP) questionnaire (49), while no predictive models of PsA development are available. A recent systematic literature review by Zabotti et al (10) supports the predictive value of various factors such severity of cutaneous involvement, psoriatic onychopathy, imaging abnormalities of subclinical synovio-entheseal inflammation, and comorbidities such as obesity or depression. If feasible, early detection and systemic treatment of these factors might reduce the PsA development. Therefore, PsO patients need to gain awareness of their disease and learn that modifying potential risk factors, such as obesity, might decrease the probability of developing PsA. The digitalization may play a role in this educational purpose. In fact, physicians and scientific societies may support public platforms for dissemination of reliable health information.

The transition phase from PsO to PsA provides a unique opportunity for early intervention (and possibly even prevention) in a population at higher risk of developing arthritis, in which a rheumatological monitoring or disease interception with therapy directed at PsO, but also PsA could be envisaged (7). The growing number of biological drugs and other molecules that act both on skin and joints in psoriatic disease might give the possibility to prevent arthritis evolution (25). It is described in literature that a very early disease interception and the appropriate psoriasis treatment may lead to the decline in skin symptoms, pain, and subclinical inflammation, as reported in the IVEPSA study (50, 51).

The widespread use of biologic therapy available for PsO may reduce the incidence of PsA. The idea to prevent PsA by early interference with the process of psoriatic disease is both fascinating and challenging, because the feasibility of such a concept mainly depends on patient selection (i.e., those with highest risk, with awareness of the at-risk situation and with sufficient compliance), and in treatment choice given that therapy should interfere with the immunological processes that promote the transition from PsO to PsA.

Digital technology could also play a role in the assessment of treatment adherence in patients with severe PsO. Recent studies suggest that apart from a one-time education on disease and therapeutics, continuing information and support may lead to better drug-adherence. Telehealth measures have shown to be beneficial for drug-adherence in patients with osteoporosis and rheumatoid arthritis (RA) (52, 53), when delivered by members of the health care team. Digital reminders, via applications or websites, may be a more feasible alternative to phone calls or mail and could show similar efficacy, especially when well designed and integrating offers such as social support sections or gamification elements (54). Of interest is the recently developed reusable electromechanical self-injection device (e-Device ava^®^) for treatment administration of certolizumab (55). Tailoring self-injection devices to individual patient preference may improve adherence to treatment and help the patients in remembering the date of self-injections (55, 56). Furthermore, e-Device aims to minimize the patient needle phobia, and the concern about safety in the treatment administration, giving the patient an electronic device that provide the administration (57). Some patients reported a higher satisfaction, self-confidence, safety, and feasibility of e-Device, compared with pre-filled pen and syringes (55, 58, 59). The use of e-Device could both assess the adherence to treatment, enable patients to track their own data, perform self-assessments, and deliver questionnaires. Ideally, e-Device would be connected with smartphones and would ask patients to complete validated questionnaires (such as patient reported outcomes (PROs), health assessment questionnaires, self-evaluation) at certain time points (such as after 1, 3 and 6 months after treatment initiation), ensuring a remote follow-up. The enthusiasm toward the use of mobile and wireless technology to support the health objective is however overshadowed by the fact that most of the available smartphone applications lack quality and scientific accuracy (60–62).

In literature, there are reports about the correlation of higher patient involvement with better adherence to therapy (63). Besides, the patient's perspective of disease state and burden is increasingly recognized as fundamental for a satisfying treatment (64). PROs are subjective measures (65) that can facilitate the assessment of physical and psychological functioning. The role of PROs is to capture patient's perspective, to provide a complete picture of the disease and, when used effectively, to aid to the holistic management of PsO patients (66). Creating applications that both patients and physicians can use to collect validated outcome parameters, such as PROs, clinical and laboratory markers is a big opportunity to improve care. An obvious advantage is a deeper involvement and understanding of patients in their treatment plan. A modern program with an easy-to-use interface, may further simplify the patient visit altogether. Patients with diabetes mellitus nowadays have blood sugar sensors that send data directly to a cloud-based application that can be seen by a physician anytime by logging into a website. Diagrams such as line-charts for disease activity outcomes, with visual information when treatment changes were performed and would be a great way to facilitate a rheumatological visit and have enormous advantages for data collection for research purposes.

The use of step counters and smartwatches may also introduce health benefits. Wearable technology includes various devices (step counters, sleep monitors etc.) and some wearable sensors have already been experimented to monitor energy consumption, step count and hand mobility. Thus, they could be used by patients and clinicians for diagnosis, follow-up and self-monitoring (67, 68). The majority of studies are on RA, but this technology could be also implemented in the management of PsA and spondylarthritis patients, as well (69). The obtained data could be integrated in the patient health record, aiming to assist clinicians in the decision-making (70). At present, this technology is integrated into smartphones, smartwatches, and other devices (such as an electromechanical self-injection devices). It needs to be simple enough for patients to be used in their daily routine, thus the design usually exploits wireless or Bluetooth technology to share information between sensors and devices. In PsO, remote questionnaires may offer a tight control, promote patient education, help identify psoriasis patients at higher risk of developing arthritis, and thus increase the number of early PsA diagnoses.

In the Italian city of Udine, we created a project, called PSOART. It was designed to guarantee a tight control and follow-up of PsO patients. It contributes to the identification of subjects at increased risk of developing arthritis through the use of questionnaires and self-evaluation. The idea behind PSOART was to empower the patients: they become the main character in their journey and not only a passive spectator of their own disease. Furthermore, PSOART may reduce the diagnostic delay and maximize the “capacity” of the rheumatologic center in screening for predictors of psoriatic arthritis development. The patient periodic assessment remains crucial and the PSOART questionnaire may be beneficial in this regard.

**Figure 2:**
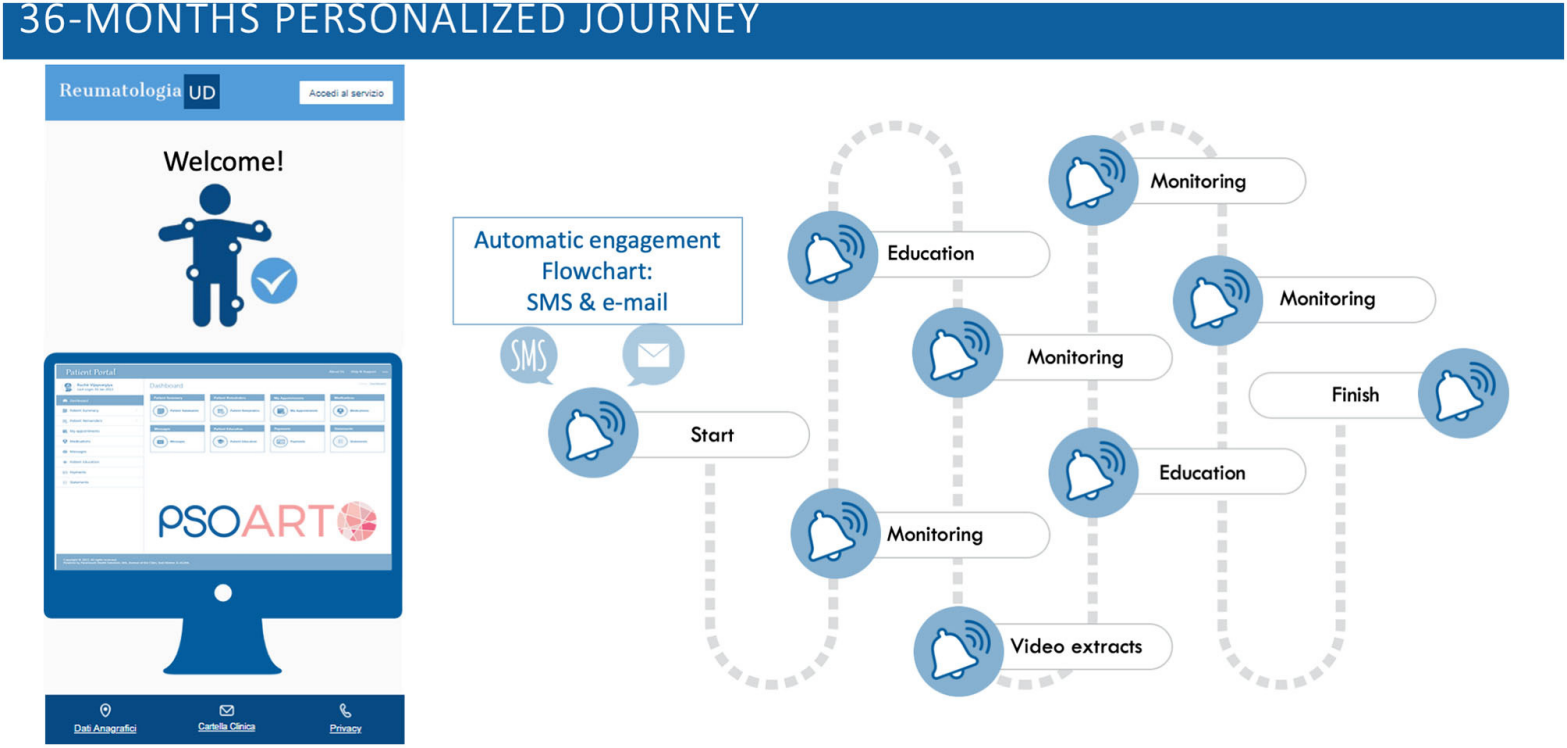
PSOART personalized journey. The 36-months patient-targeted journey is hereby represented. The PSOART platform uses an automatic email/sms communication system targeted on the patient. It offers encouraging goal, such as educational material and video extracts, as well as “alert” for the rheumatologic center [https://www.psoart.com].

To make PSOART easy to use and feasible, the questionnaire was digitalized for use on internet platforms and smartphone applications. The questionnaire and self-evaluation chosen were validated in the literature (such as, PSAID, BASDAI, HAQ questionnaire, Visual Analog Scale for Pain etc.) and adapted to the digital format. The patient will login to the platform and complete the self-assessment questionnaires at pre-established time points (baseline, 1, 3, 6 months etc.) over the span of 3 years and share the results with the physician (Figure 2).

During the patient journey, the platform will also provide educational and informational material, such as video-interviews and written articles, regarding lifestyle, tips on disease management and details about treatments. The educational material will be evaluated and approved by clinicians and aims to minimize “fake news” in rheumatology. The PSOART platform allows remote monitoring and disease interception in patients affected by PsO and seems to be well accepted by patients, as suggested by the positive comments and feedbacks. Hopefully, PSOART would not only optimize the patient follow-up, but also allow the creation of a predictive score for the development of PsA, opening the way to a possible prevention of PsA.

In addition, electronic health records (EHR), the digital version of a patient's paper chart, could ideally become the standard of practice for record keeping. EHR has the potential to help in the management of chronic and heterogeneous disease. EHR could alert dangerous drug-to-drug interactions or help with imaging assessment, depending on the disease and guidelines. In the USA, some researchers used EHR to alert clinicians that a patient using an immunosuppressive drug should receive an influenza vaccine. This intervention increased the influenza vaccination rate from 47 to 65% (71). Furthermore, EHR could integrate and organize information from other digital sources, such as wearable devices and apps, providing important opportunities to collect data for research purposes.

### PsO Patients at Higher and Imminent Risk of Developing Arthritis

PsO patients at higher and imminent risk of developing PsA are patients presenting musculoskeletal complaints (e.g., arthralgia, achilles' tendon pain) or fatigue (5, 7, 22). In line with this, Eder et al., showed that arthralgia in psoriasis females is a strong predictor of PsA development (9), and Zabotti et al., described that psoriatic patients with arthralgia (PsOAr) were more prone to develop PsA compared to psoriatic patients without musculoskeletal complaints (PsO) (7). These data highlight that patient-reported symptoms in the preclinical phases of PsA may be a marker of imminent PsA development. However, the prodromal phase is difficult to define due to non-specific musculoskeletal symptoms (such as joint pain, fatigue and stiffness) that can also be caused by other disease mimicking PsA (such as concomitant osteoarthritis, fibromyalgia, or chronic pain (6, 72, 73). Therefore, remote monitoring and self-assessment cannot replace face-to-face visits. While monitoring visits using telehealth revealed promising results in patients with longstanding rheumatological diseases, the detection of definite signs of prodromal PsA requires a face-to-face visit. A clinical exam is necessary to assess PsA risk factors including tender and swollen joint counts. While tenderness can be assessed by patients themselves quite well, studies show discrepancies between the number of swollen joints assessed by patients and rheumatologists (74, 75).

Moreover, the in-person visit are viewed by some clinicians as a method for ensuring compliance to treatment and generate confidentiality of the doctor-patient relationship (76). The loss of physical contact and the difficult emotional relationship between clinicians and patients have been reported as the major limitation of telemedicine (77, 78). Upcoming EULAR points to consider on remote care do therefore reinforce that telemedicine should be seen as an additional tool, rather than a substitute for a face-to-face visit.

Digitalization may contribute to the self-assessment, allowing patients to quickly report the onset of symptoms, assumed joint swelling, and other changes (such as dactylitis). PROs for inflammatory arthritis have been developed and validated to correlate with clinical measures of disease activity (79). The assessment of web-based PROs and paper-based PROs in psoriatic arthritis proved to be comparable (80), opening the way to patient self-monitoring and in particular to a remote collection of questionnaire (81). Besides, some studies describe a good feasibility for remote monitoring of skin disease in PsO patients (36) and for patient education (82). Therefore, patients with chronic conditions may take advantage in self-management, to bring the patient in the center of his own journey. The patient role in the improvement of the health quality is crucial, in fact people living with rheumatic and musculoskeletal diseases may highlight the most important symptoms, making a difference for disease management. With this aim, digital technology in the form of programs and devices promotes healthy behavior and encourage patients to take health-related actions (29). Theoretically, the spreading and accessibility of digital technology may enable researchers to collect health-related data more frequently and may help to build-up patient networks in communities. The creation of such digital platforms, however, is still challenging (29).

Recurrent self-assessment could be crucial to deliver remote questionnaire and record data by applications. Frequent patient monitoring is an integral part of the treat-to-target strategy enabling early detection of disease flares. Telemedicine and remote monitoring may be convenient for many patients with impaired mobility and may reduce the number of visits to the rheumatologist office. The remote self-assessment would also lead to identify and screen PsO patients at risk of developing arthritis, targeting an early identification of early onset of musculoskeletal (MSK) pain such as suspected arthralgia. Subsequently, the patient could be evaluated by the rheumatologists in the setting of “telerheumatology”. The target would be to screen the MSK symptoms and differentiate mechanical from inflammatory pain. It is also necessary to take care of the axial involvement in PsA, a frequent and underdiagnosed feature (39), unlikely assessable in a remote visit. Therefore, in the setting of a suspected PsA, the rheumatologists could recommend both blood chemistry tests and imaging evaluation even before the clinical assessment, aiming to an accurate and early diagnosis in the first face-to-face medical appointment.

In the setting of patient at higher and imminent risk of developing PsA, AI-based tools (symptom checkers) are designed to collect patient symptoms, determine possible causes and direct the patient to the right specialist. In rheumatology, symptoms checkers could ensure a quick referral to the specialists and reduce diagnostic delay (14). Recently, a preliminary smartphone sensors-based measurement tool was tested in PsA (83). The smartphone gyroscope, accelerometer and camera were used to assess the arm joint function, the leg joint function, and the dactylitis and nail involvement, respectively. The authors reported that the application could distinguish some clinical features of PsA that might be helpful to early detect high risk factors for PsA development.

AI has also been tested in RA for prediction of disease progression, flares and mortality (84, 85), and in osteoarthritis for image recognition, helping in the interpretation of musculoskeletal pathologies (86). These tools are still rudimentary, nonetheless they have the potential to assist clinicians in therapeutic decisions. For an harmonization of digital apps, EULAR has recently published points to consider for the development, evaluation and implementation of mobile health applications (87), aiming to provide guidance and guidelines on important aspects of self-management in patient with rheumatic and musculoskeletal diseases (88). Although digitalization in rheumatology has great potential, attention should be paid to health equity, as the most vulnerable patients may lack the resources required for a telemedicine visit or remote self-assessment devices (89), and to the risk of medicalization when applying digital health instruments in a heterogeneous disease (90).

## Conclusion

The transition phases from PsO to PsA require better characterization. Thus, a large amount of data from rheumatological services is needed to perform epidemiologic analysis. Collecting PROs via digital support provides a new tool for the monitoring of psoriatic disease. Moreover, the assessment of PROs via an online application may allow to intercept PsO patients at increased risk of transition to PsA, who otherwise would go unnoticed.

Modern models of care for PsO and PsA highlight the importance of patient involvement and self-management (91). In this scenario, integrating digital health technology will offer opportunities to complement rheumatology care, even beyond a global pandemic.

## Author Contributions

IG and AZ contributed to conception and design of the study. IG wrote the first draft of the manuscript. All authors contributed to manuscript revision, read, and approved the submitted version.

## Conflict of Interest

The authors declare that the research was conducted in the absence of any commercial or financial relationships that could be construed as a potential conflict of interest.

## Publisher's Note

All claims expressed in this article are solely those of the authors and do not necessarily represent those of their affiliated organizations, or those of the publisher, the editors and the reviewers. Any product that may be evaluated in this article, or claim that may be made by its manufacturer, is not guaranteed or endorsed by the publisher.
